# Capillary and van der Waals interactions on CaF_2_ crystals from amplitude modulation AFM force reconstruction profiles under ambient conditions

**DOI:** 10.3762/bjnano.6.84

**Published:** 2015-03-25

**Authors:** Annalisa Calò, Oriol Vidal Robles, Sergio Santos, Albert Verdaguer

**Affiliations:** 1 Institut Català de Nanociència i Nanotecnologia (ICN2), Campus UAB, Bellaterra, Barcelona, 08193, Spain; 2 Departament de Disseny i Programació de Sistemes Electrònics, Universitat Politècnica de Catalunya (UPC), Av. Bases 61, Manresa, Barcelona, 08242, Spain; 3 Consejo Superior de Investigaciones Científicas (CSIC), ICN2 Building, Bellaterra, Barcelona, 08193, Spain

**Keywords:** amplitude modulation (AM) AFM, dynamic capillary interactions, dissipative nanoscale interactions, CaF_2_ wetting, force reconstruction

## Abstract

There has been much interest in the past two decades to produce experimental force profiles characteristic of the interaction between nanoscale objects or a nanoscale object and a plane. Arguably, the advent of the atomic force microscope AFM was instrumental in driving such efforts because, in principle, force profiles could be recovered directly. Nevertheless, it has taken years before techniques have developed enough as to recover the attractive part of the force with relatively low noise and without missing information on critical ranges, particularly under ambient conditions where capillary interactions are believed to dominate. Thus a systematic study of the different profiles that may arise in such situations is still lacking. Here we employ the surfaces of CaF_2_, on which nanoscale water films form, to report on the range and force profiles that might originate by dynamic capillary interactions occurring between an AFM tip and nanoscale water patches. Three types of force profiles were observed under ambient conditions. One in which the force decay resembles the well-known inverse-square law typical of van der Waals interactions during the first 0.5–1 nm of decay, a second one in which the force decays almost linearly, in relatively good agreement with capillary force predicted by the constant chemical potential approximation, and a third one in which the attractive force is almost constant, i.e., forms a plateau, up to 3–4 nm above the surface when the formation of a capillary neck dominates the tip–sample interaction.

## Introduction

The study of the forces and energies released when a nanometric tip and a surface are progressively brought into contact has driven much of the recent investigation in atomic force microscopy (AFM) and has allowed for the mapping of materials properties while scanning [1–3] besides finding optimal imaging conditions [4–5]. Furthermore, understanding and distinguishing among relevant interactions in a broad range of materials at the nanoscale and in different environmental conditions is important from the fundamental point of view [6–7]. This is particularly true when working in air under ambient conditions, where the presence of thin layers of water is ubiquitous even on highly hydrophobic surfaces [8–10] and specific interactions (hydration, capillary forces) [11] need to be accounted for, which can be effective at relatively large tip–sample distances [12–13] and can exhibit unexpected distance dependencies [14].

Contact AFM measurements, in which the force is determined from the static deflection of the cantilever during approach [15], can readily record the tip–sample interaction force and have been used extensively to characterize a variety of nanoscale materials, from soft biomaterials (vesicles, viruses) [16–17], to organic thin films [18–21] and self-assembled monolayers [22] in liquid and in air, especially at those short separations where breakthrough events and sample mechanical deformations occur. However, in such experiments, the jump-to-contact instability [6–7] screens even strong (van der Waals, capillary) [13,23] interactions and actually prevented the experimental access to that region in force curves where attractive forces dominate. This instability has been especially observed when working in air and when soft cantilevers were employed to increase the sensitivity [6–7,15,24].

Dynamic modes proved to overcome the limitations of contact measurements in detecting attractive forces [11,25–27]. In these modes the cantilever is mechanically driven at a fixed oscillation frequency and interaction forces can be determined through analytical descriptions [28] or numerical methods [29] from minute changes in the amplitude [30] or in the frequency [6–7] of the oscillation when the tip–sample separation distance is changed. Furthermore, the advantage of performing dynamic AFM measurements, as for example amplitude modulation AM-AFM measurements, is that experimental observables, i.e., the phase lag of the cantilever relative to the driving force, can be directly related to the energy dissipated in the tip–sample interaction [31–33]. Identifying and separating individual contributions to the net energy dissipation by their physical origin and/or distance-dependence [34] has been the object of recent efforts in the direction of performing quantitative measurements with the AFM [35–36]. For example, Gadelrab and coauthors showed that the difference in the phase signal compared to a purely conservative attractive interaction (ΔΦ) can be used to distinguish between dissipative processes (hysteresis, viscosity) occurring in non-contact [37].

In this work, we used the Sader–Jarvis–Katan method [38–39] to reconstruct force vs minimum distance of approach (*F*_ts_ vs *d*_min_) curves [24] from amplitude and phase distance (APD) curves collected on CaF_2_ crystals containing water patches on their surface. We then compared the obtained profiles with those observed under low humidity conditions, i.e., when water is not present on the crystals. The Sader–Jarvis–Katan method has been recently applied to reconstruct the force on mica and graphite samples and changes in the shape of the resulting profiles were studied under different environmental conditions. An evolution in the force curves was observed keeping these surfaces at high humidity levels for long times [9–10]. At high humidity and long times of exposure plateau-like features where force was approximately constant for 1–2 nm before contact were observed. This kind of force profile, approximating a square well [14], has been already proposed to explain, from the phenomenological point of view, typical APD curves observed in dynamic AFM and interpreted as the result of dynamic capillary interactions. In the experiments reported on graphite additional spectroscopic IR measurements were performed [9–10] to exclude that the observed change in force profiles could depend on chemical contamination or aging of the sample. For example, carbonates could be present on the surface due to the exposure to a set of environmental conditions for prolonged times. Although IR measurements seemed to prove an increase in the intensity of the peaks related to water with time, in the AM-AFM experiments water was not visualized on the surface, making it impossible to distinguish between dry, wet or contaminated regions of the sample or to have an estimation of the thickness of the water layer adsorbed on the surface.

In the case of CaF_2_, well-defined water patches on the crystals surface can be induced by controlling the environmental humidity [40], which are recognizable through AM-AFM images [41–42]. Thus, direct correlations between the reconstructed force profiles and topographical data are possible in situ and the evolution of force curves during the patch formation can be followed in short time experiments, thus limiting sample contamination. In these crystals we unambiguously identified fingerprints of capillary interactions, i.e., their onset and distance dependencies, from force curves on top of the water patches and from the simultaneous observation of both the corresponding dissipated energy, calculated according to the Cleveland equation [31], and the ΔΦ vs distance evolution [27]. Our results indicate that standard expressions for capillary forces based on a constant chemical potential can also be a valuable tool to predict the experimental phenomena observed in dynamic AFM [14].

## Results

**Figure 1:**
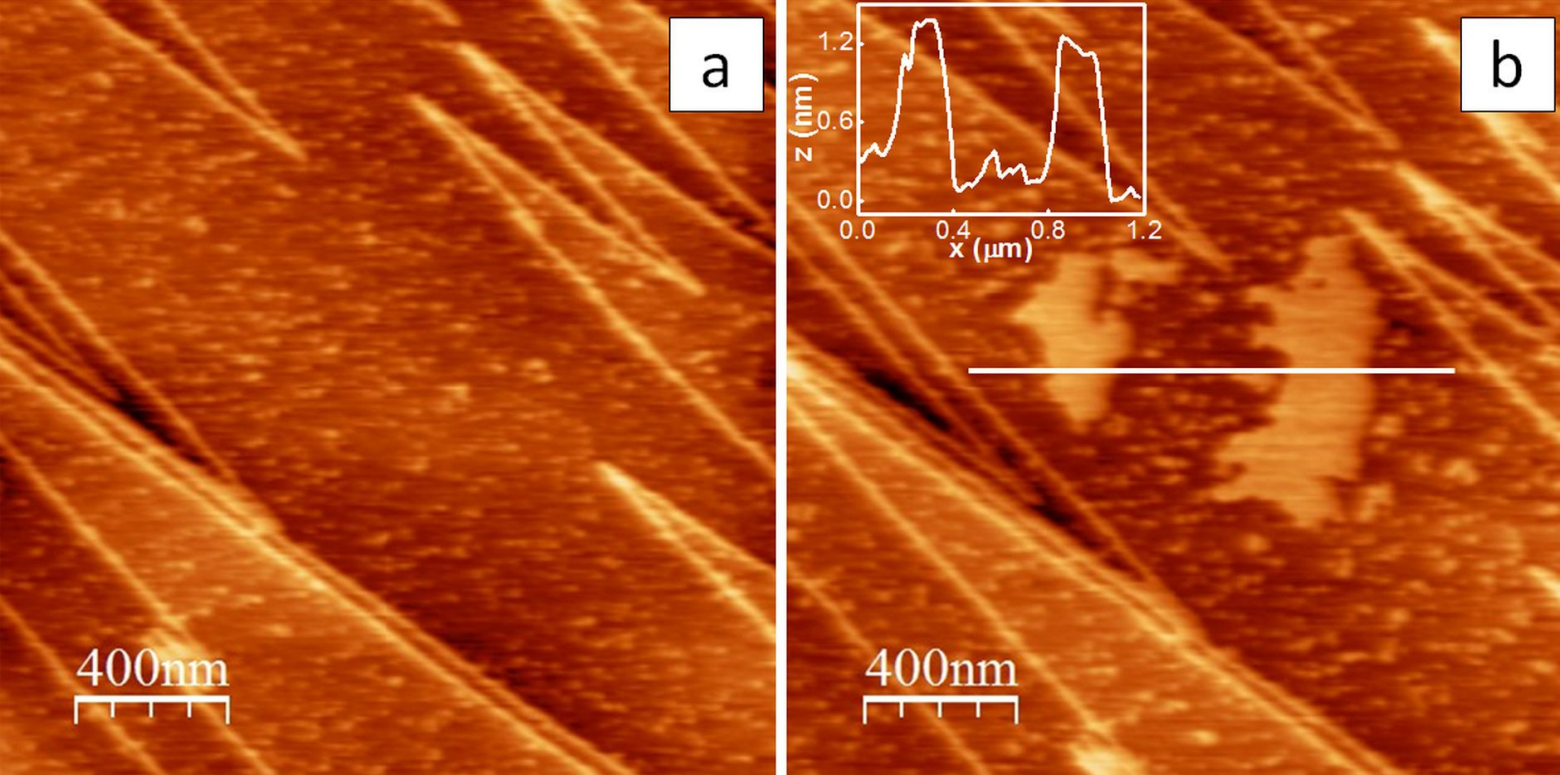
(a, b) AM-AFM images of a CaF_2_ crystal taken immediately after cleavage (a) and after a water patch is formed on the crystal surface (b). *Z*_scale_ is 3 nm for both images. (b, Inset). Topographic profile corresponding to the white line in the image in (b).

Figure 1a and Figure 1b show AFM images of the surface of a CaF_2_ crystal before (a) and after (b) the formation of water patches has been induced (see Experimental section). In the image in Figure 1a a micrometer-sized terrace is visible, which is delimited by triangular (V-shaped) steps. These features are characteristic of freshly cleaved CaF_2_ (111) surfaces, for which the shape of the steps (triangular or long parallel) depends on the cleavage direction [40]. Water is already present on top of the crystal surface after cleavage and accumulates in small drops about 1 nm thick, as inferred from the topographic profile of the AFM image in Figure 1a. At ambient temperature and RH (RH ca. 40%) water accumulates at the flat terraces on the crystal surface forming rounded islands [40] (see Figure 1b and Experimental section) over a time scale of few minutes. Under our experimental conditions, the islands exhibited a typical thickness of 1–1.5 nm (see the inset of Figure 1b) that did not change during experiments, i.e., by repeatedly scanning or collecting APD curves on the same area [41].

**Figure 2:**
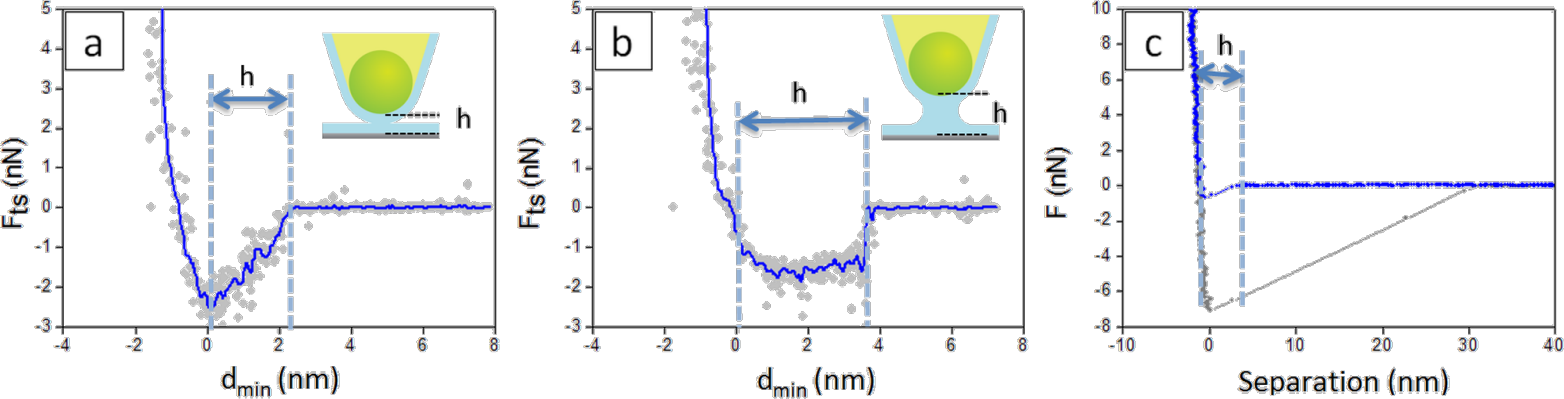
(a,b) Reconstructed force curves on top of the terrace in Figure 1a (a) and on top of the bigger water patch in Figure 1b (b); free amplitude of tip oscillation: *A*_0_ = 23 nm. Raw and smooth data are, respectively, indicated as solid circles in grey and as continuous lines in blue. (c) *F* vs separation curves acquired in contact mode on a wet CaF_2_ crystal. The extension and retraction paths are indicated, respectively, in blue and in grey only in (c).

In Figure 2a and Figure 2b typical *F*_ts_ vs *d*_min_ curves are reported, which have been reconstructed according to Equation 1 (see Experimental section) from APD curves collected respectively in the middle of the terrace in Figure 1a and on top of the bigger water patch in Figure 1b. Raw and smooth data are respectively indicated as solid circles in grey and as continuous lines in blue. Curves such as the one shown in Figure 2a exhibit an approximately linear decay in the force, starting at relatively large tip–sample distances ≥2 nm and terminating at the contact with the hard CaF_2_ surface. Curves such as the one shown in Figure 2b exhibit an abrupt jump in the long range, followed by an almost constant plateau, which spans nearly 4 nm of vertical distance. Then *F*_ts_ increases very rapidly with *d*_min_ and the force profile is similar to that observed at distances past the minima in force in Figure 2a. Here mechanical contact occurs and repulsive forces dominate the tip–sample interaction [27]. We set *d*_min_ = 0 by eye at the point where *F*_ts_ changes its slope and starts to increase rapidly, corresponding to the beginning of the mechanical contact between the tip and the surface.

We found that the distance from the decay of *F*_ts_ in the long range to *d*_min_ = 0 is 2.33 ± 0.17 nm for curves like the one shown in Figure 2a and 3.66 ± 0.20 nm for curves like the one shown in Figure 2b. A similar distance of 3.93 ± 0.40 nm from the jump-to-contact to the point of zero separation is found in the approach path of force curves collected in contact mode on CaF_2_ crystals with adsorbed water layers on top of the surface (see Figure 2c) at ca. 40% RH.

Force profiles such as the ones in Figure 2a and Figure 2b indicate that capillary interactions, i.e., intermolecular attractive forces acting between water layers present on the surface of the tip and of the crystal, take place, which involve the formation and the rupture of a capillary bridge [3,14].

A linear decay of the force vs tip–sample distance such as that shown in Figure 2a is predicted in the limit of constant chemical potential or constant vapor pressure, according to which, when the tip approaches the sample, water condensation can induce the formation of a nanometer-size water bridge [43–45]. Expressions for the capillary force (*F*_CAP_) based on these assumptions (see Equation 2 in the Experimental section) have been employed in dynamic AFM, together with more complex derivations based on the limit of a constant meniscus volume [3,14]. The approximation that assumes a constant volume of the meniscus considers that water in the capillary bridge essentially derives from water layers already present at the surface of the tip and the sample, rather than from the condensation of ambient vapor due to tip proximity. The choice of the more realistic model in specific contexts should take into account the timescale of water condensation and the tapping frequency [43–44].

The force profile shown in Figure 2b resembles force evolutions employed in the context of dynamic AFM, in which the predominantly attractive component of the net force is relatively independent on the distance and covers a range of some nanometers above the surface [14]. In Equation 3 it has been assumed that when the capillary forms the force is constant and equal to the adhesion force (*F*_AD_) (see Experimental section).

**Figure 3:**
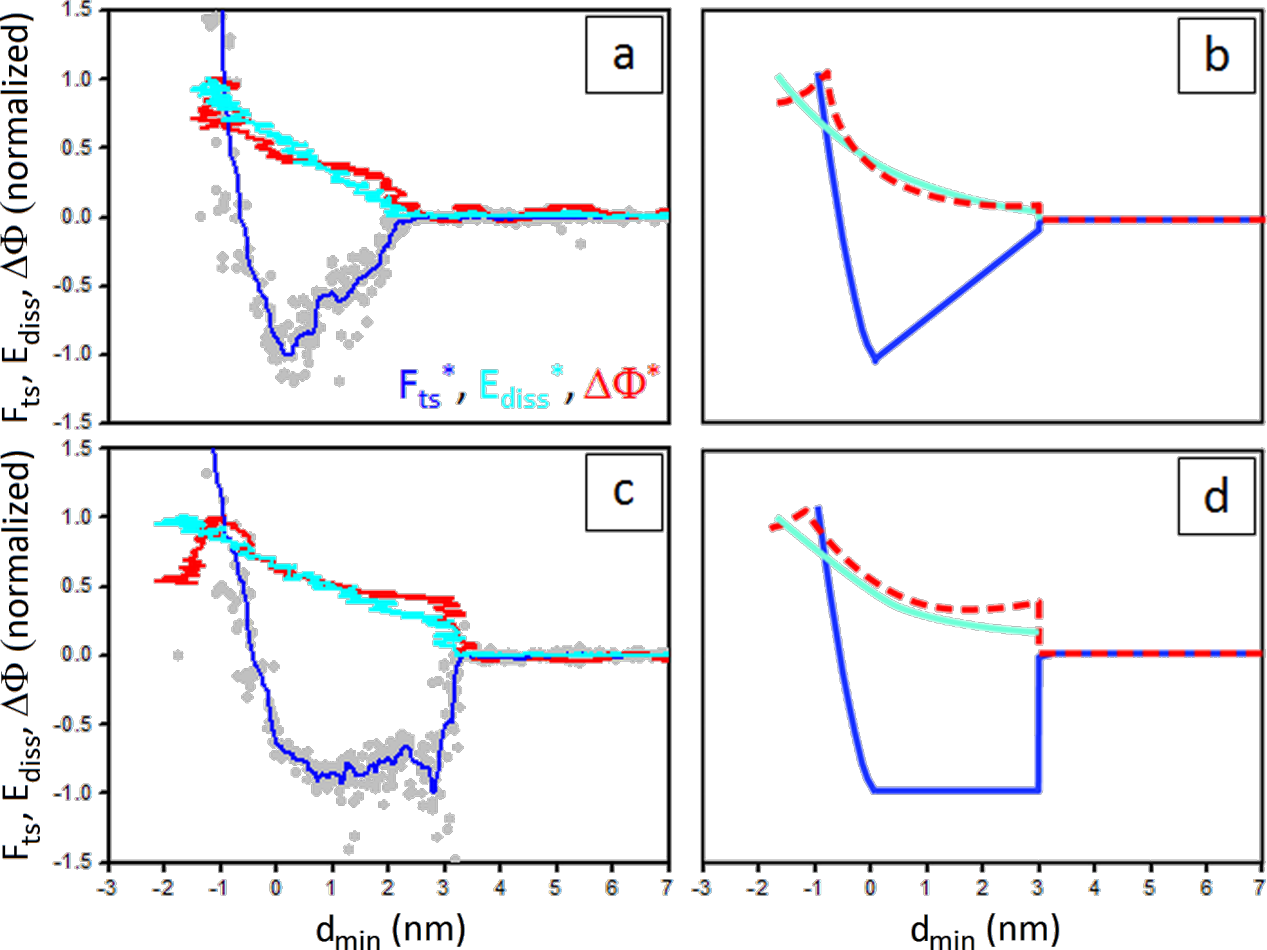
(a,c) Experimental normalized *F*_ts_, *E*_diss_ and ΔΦ curves vs *d*_min_ collected on a CaF_2_ sample containing water droplets (a) and on a water patch on top of the CaF_2_ crystal (c). *F*_AD_ = 2.8 nN, *E*_max_ = 82 eV, ΔΦ_max_ = 11.0° in (a) and *F*_AD_ = 2.0 nN, *E*_max_ = 114 eV, ΔΦ_max_ = 13.9° in (c) (see main text). *A*_0_ in (a) and (c) is 23 nm. (b,d) Simulated normalized *F*_ts_, *E*_diss_ and ΔΦ curves in which the capillary force is expressed by Equation 2 (b) and Equation 3 (d). The forces and parameters employed in the simulation are described in the Experimental section. *F*_ts_^*^, *E*_diss_^*^ and ΔΦ^*^ are respectively indicated in blue, cyan and red.

In this work, Equation 2 and 11 have been employed to reconstruct force curves according to the Sader–Jarvis–Katan formalism in numerical simulations (see Experimental section). The results of the simulations are shown in Figure 3, in which simulated *F*_ts_ curves (Figure 3b and Figure 3d) are compared with the experimental curves (Figure 3a and Figure 3c).

Forces such as those in Equation 2 and Equation 3 suppose a *d*_on_/*d*_off_ mechanism. They act at a distance *d* < *d*_on_ on tip approach and at *d* < *d*_off_ on tip retraction, with *d*_off_ ≥ *d*_on_ [14]. The difference between these two distances leads to hysteresis in the long range. That is, as the tip approaches the sample a capillary bridge forms at *d* = *d*_on_, which ruptures on tip retraction at *d* = *d*_off_. If the distances of formation and rupture of the capillary bridge do not coincide, i.e., if *d*_off_ > *d*_on_, hysteresis occurs and energy is dissipated in the interaction. If *d*_off_ = *d*_on_ there is no hysteresis and the interaction is conservative [14].

In the reconstructed force curve of Figure 2a a step-like discontinuity that could be associated with the formation of a capillary bridge is not distinguishable. Instead, a slow decay is observed in the force at a distance, 2.33 nm, that approximately matches the sum of the height of the absorbed water droplets on top of hydrated CaF_2_ and the expected thickness of the water layer on top of the tip (1 nm at room temperature, RH ca. 40% and in the absence of contamination) [46]. This distance indicates that water meniscus forms upon geometrical contact of the absorbed water layers on top of the tip and the crystal (see the sketch in Figure 2a) or that capillary condensation due to tip–sample proximity or other instabilities (for example, van der Waals interactions) can be considered negligible in this case [45]. Alternatively, the measured distance could point out that water layers on the tip and the sample simply overlap on approach, i.e., a capillary neck does not form at all in this case, and the decaying region in the force marks the tip penetration into confined water, a scenario that seems also plausible in dynamic AFM [14].

When a continuous water patch is present on top of the CaF_2_ crystal, a linear increase in the attractive force was still observed for some reconstructed force curves but a different feature appeared regularly. In most of the force curves an abrupt drop followed by a constant force plateau was observed (Figure 2b), similarly to what reported for graphite exposed to high humidity for long times [9]. The abrupt drop occurs at a distance of 3.66 nm that approximately corresponds to three times the thickness of the water islands as measured by AFM topography. This number is in impressive agreement with *d*_on_ = 3*h*, with *h* being the height of adsorbed water layers on the hydrated surfaces of the tip and the sample (here *h*_tip_ ≈ *h*_sample_) and it is expected when instabilities due to van der Waals attraction and/or capillary condensation take place under approach [14,44]. Interestingly, we found *d*_on_ = 3*h* only when stable water patches were present on the surface, a condition that is obtained experimentally by leaving the ambient humidity spontaneously increase from 10% to the ambient value of 40% after sample preparation (see Experimental section). This increase of RH should reduce the free energy barrier for water nucleation, thus increasing the water condensation rate [3]. It is worth to point out that force vs distance evolutions changing from almost linear decays to abrupt jumps followed by a constant plateau have been shown in function of the increasing relative vapor pressure in Monte Carlo simulations of the interaction forces between nanoparticles [13] and between a rigid nanoparticle and a flat plate [47].

Finally notice that, due to the imaging conditions (see Experimental section), the apparent height of the water droplets/layers measured by AFM topography (Figure 1a and Figure 1b) should be equal or close to the true value as water perturbation due to mechanical contact is avoided [41–42].

## Discussion

The different dynamic interactions in the two situations depicted in Figure 2a and Figure 2b is corroborated by the analysis of the normalized dissipated energy and phase difference evolutions vs *d*_min_ in the long range (see also Equation 4 and Equation 5 of the Experimental section). Here, the asterisk indicates normalization, *E*_diss_^*^ = *E*_diss_/*E*_max_ and ΔΦ^*^ = ΔΦ/ΔΦ_max_, with *E*_max_ and ΔΦ_max_ corresponding to the maxima in the approach curve [27]. This is shown in Figure 3, in which the reported force curves are also normalized, i.e., *F*_ts_ is divided by the minimum force *F*_AD_.

The simulated curves in Figure 3b and Figure 3d reproduce very well the experimental observations Figure 3a and Figure 3c. When a long range hysteretic force with a *d*_on_/*d*_off_ mechanism such as the one described in Equation 2 is activated in the simulation (here *d*_on_ = 3 nm and *d*_off_ = 3.3 nm), a small step-like discontinuity is observed at *d* = *d*_on_ in the *F*_ts_^*^, *E*_diss_^*^ and ΔΦ^*^ signals (see respectively the blue, the cyan and the dashed red lines in Figure 3b). This effect becomes more pronounced when a capillary force such as the one described in Equation 3 is employed (see the blue, cyan and dashed red lines in Figure 3d, here being *d*_on_ = 3 nm and *d*_off_ = 5 nm) [14]. The jump in *E*_diss_ should correspond to the difference between the area of the approach and retraction force curves used in the simulation [27].

Notice that the force reconstruction process according to the Sader–Jarvis–Katan formalism recovers the conservative tip–sample force and fails to record events that occur at distances larger than *d*_on_ [27]. Thus, the footprints of a *d*_on_/*d*_off_ dissipative mechanism result in the observed steps in the *E*_diss_^*^ and ΔΦ^*^ signals [27,37].

In experimental *E*_diss_ and ΔΦ vs *d*_min_ curves collected on water patches on CaF_2_ we measured, respectively, a step of (25.17 ± 4.82) eV and of (5.52 ± 0.70)° in correspondence of the abrupt drop in force (see Figure 3c). The value obtained for *E*_diss_ is in the expected range (20–50 eV) for the formation and the rupture of a capillary neck by means of tips with a radius of about 20 nm [3,45]. For curves collected under low humidity conditions (see Figure 3a) *E*_diss_ did not show any step-like discontinuity, while ΔΦ exhibited a smaller step (3.78 ± 0.75)°. Both signals indicate that a lower amount of energy is dissipated in the process of forming and breaking the capillary bridge in this case or, equivalently, that the distances of formation and rupture of the capillary bridge are similar.

**Figure 4:**
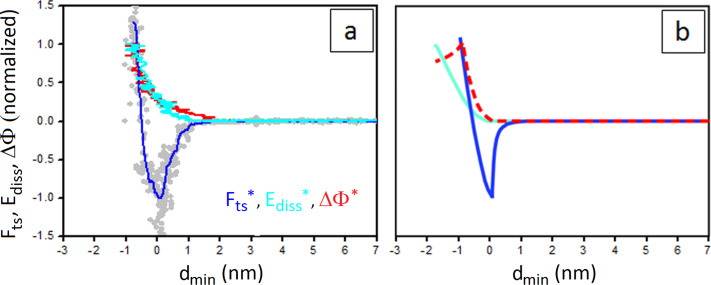
(a) Experimental *F*_ts_^*^, *E*_diss_^*^ and ΔΦ^*^ curves vs *d*_min_ collected on a dry CaF_2_ sample. *F*_AD_ = 11.4 nN, *E*_max_ = 418 eV, ΔΦ_max_ = 12.3°; *A*_0_ = 80 nm. (b) Simulated *F*_ts_^*^, *E*_diss_^*^ and ΔΦ^*^ curves. For the forces and parameters employed in this case in the simulation see Experimental section. *F*_ts_^*^, *E*_diss_^*^ and ΔΦ^*^ are indicated as blue, cyan and red lines, respectively.

In Figure 4 normalized force curves reconstructed on CaF_2_ crystals in dry conditions (Figure 4a), are compared with the results of numerical simulations (Figure 4b). Both panels include *E*_diss_^*^ and ΔΦ^*^ signals. Force curves reconstructed from experiments in dry conditions, i.e. after incubating CaF_2_ crystals for 30 minutes at 120 °C under N_2_ flow before measurements, usually show a well-defined curvature around *d*_min_ ~ 1 nm from contact, resembling an inverse-square law decay (see the blue line in Figure 4a). At this distance, *E*_diss_^*^ and ΔΦ^*^ start to increase continuously with diminishing *d*_min_ (see the lines in cyan and in red in Figure 4a). The simulated curves show a similar behavior (Figure 4b).

**Figure 5:**
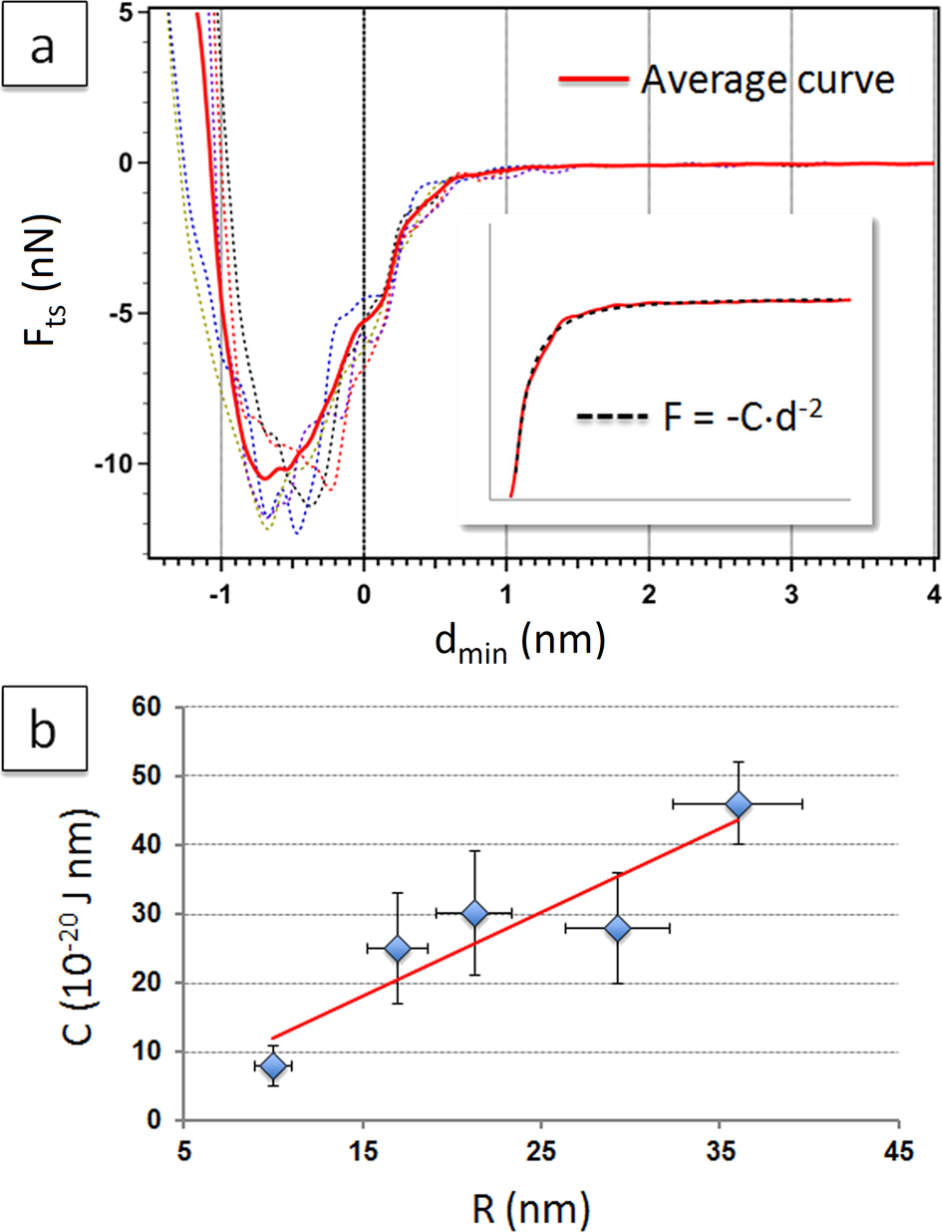
(a) Experimental (dotted lines) and average (red line) *F*_ts_ vs *d*_min_ curves collected on a CaF_2_ crystal in the absence of water droplets and/or patches (*A*_0_ = 80 nm). (a, Inset) Detail of the average curve in the region 0.2 nm < *d*_min_ < 1.8 nm and corresponding fit according to Equation 6. (b) Dependence of the fitting parameter C on the tip radius. A linear fit of the data is indicated in red.

In Figure 5a five different reconstructed *F*_ts_ vs *d*_min_ curves, represented as dotted lines, are shown which are taken in the same region of a dry CaF_2_ crystal. The strong adhesion force (*F*_AD_ ≈ 10 nN) depends on tip radius (*R* ≈ 40 nm in this case, see Supporting Information File 1, Figure S2).

Curves were aligned and averaged before fitting in order to minimize noise in the region of interest. Alignment of individual curves at *d*_min_ = 0 in the graph was established according to the distance at which discontinuities, in the form of changes of slope, were observed in the *E*_diss_^*^ and ΔΦ^*^ signals vs *d*_min_ (data not shown). As it can be observed in Figure 5a, the force around *d*_min_ = 0 contains an inflection point, or a shoulder. For some curves this point was very clear, while for other curves we found its position quite arbitrary to determine in a range of ± 0.4 nm (see also Supporting Information File 1, Figure S2).

The average force curve, shown as a continuous line in red in Figure 5a, was fitted with a power law decay [48]:

[6]
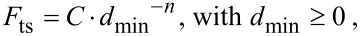


leaving *C* and *n* as free parameters. In general, good fittings were obtained with *n* = 2, according to the χ^2^ values (see the inset of Figure 5a). In Supporting Information File 1, Figure S3, the effect of a slight modification of the point at zero distance in the average curve on the fitting results is reported.

Obtained values for *C* from the fitting were stable in the same experiment and in different experiments with tips of similar radius. The fitting parameter *C* showed a strong dependency on the tip radius. In Figure 5b its average value is reported as a function of the tip radius *R*, determined from the onset of the smooth transition between the attractive and the repulsive regime (see Experimental section). By using this method, we estimated an error of ca. 20% for the tip radius [49]. The experimental *C* vs tip radius values adapt to a linear tendency with a coefficient of determination of *R*^2^ = 0.84.

Based on these results, the observed force could be explained as a pure van der Waals attractive force operating between the tip and the CaF_2_ crystal according to Equation 7 [27,50]. From the fitting of the linear tendency, and according to the simplified model of van der Waals interactions used in Equation 7, we estimated a value for the Hamaker constant of (7.3 ± 0.4) · 10^−20^ J. Although this value has not to be taken as an accurate measurement of the Hamaker constant, it is in the range of the experimental and theoretical values reported in the literature for the CaF_2_/SiO_2_ pair [50].

## Conclusion

Force vs distance curves were collected in both contact mode AFM and reconstructed from dynamic AM-AFM experiments on CaF_2_ crystals exhibiting water patches on the surface. Results indicate that dissipative processes occur that involve the formation and the rupture of a capillary bridge. This can be inferred from the high hysteresis in the retracting portion of the static force curves, which spans an average distance of about 30 nm and exhibits an adhesion force of almost 6 nN compared to the approach path. But it is only from dynamic AFM experiments that interactions in the long (attractive) range can be truly probed and their effect on the energy stored by the cantilever during interaction with the sample accurately measured. In this work, two different interaction regimes have been identified in the force vs minimum tip–sample separation distance in the attractive range, in which (a) the force decreases almost linearly with separation or (b) exhibits an abrupt drop followed by a constant plateau. These regimes, which are indistinguishable in contact mode experiments, have been here related to the occurrence of dynamic capillary interactions during tip approach when relative humidity is allowed to spontaneously reach the ambient value of about 40% and homogeneous micrometer-sized water patches stabilize on top of the crystals surface. The dissipation induced by the capillary bridge formation is unambiguously identified in experimental *E*_diss_ and ΔΦ curves and force profiles are compared with simulations in the long range, where the contact between water layers on top of the tip and the surface occurs. CaF_2_ is demonstrated to be an ideal surface to probe the presence of these interactions, as it allows correlating directly force profiles and topographical data from AFM images and distinguishing force profiles in wet and in dry samples. Under dry conditions, van der Waals interactions seem to be the main contribution to the net tip–sample attractive force.

## Experimental

### Materials and Methods

All the experiments were performed using an Asylum Research AFM MFP-3D microscope equipped with a cooler/heater sample stage and operating in AM mode. APD curves (rate: 1.5 Hz) were collected on top of CaF_2_ crystals at free amplitudes above the critical region of bi-stability [51–52], in order to achieve a smooth transition from the attractive to the repulsive regime as the amplitude is reduced to approximately 10% of its free value in a distance range of 15 nm. In this way, force profiles can be reconstructed in the whole range of distances, from long range interactions to tip–sample mechanical contact, without discontinuities [9,27]. Cantilevers with resonance frequency *f*_0_ ≈ 300 kHz, spring constant *k* ≈ 45 N/m and nominal tip radius *R*_0_ ≈ 10 nm were employed (Nanosensors PPP-NCHR), for which avoiding discontinuity requires a free amplitude *A*_0_ ≈ 23 nm. The conversion of the amplitude in volts to amplitude in nm was determined by adjusting the tilt of *d*_min_ vs *z*_c_ curves in the repulsive region till a flat plateau was obtained [34]. The resonance frequency, spring constant and quality factor (*Q* ≈ 400) of the cantilever were calibrated in situ at a distance smaller than 200 nm from the surface. The resonance frequency was found to decrease approximately 30 Hz with respect to the calibrated value at a few micrometers above the surface. The tip radius *R* was constantly monitored in situ as well, by checking that the onset of the smooth transition remained constant during experiments [49]. When free amplitudes *A*_0_(*R*) higher than 23 nm were employed, the tip radius was estimated to be *R* = *R*_0_ · (*A*_0_(*R*)/*A*_0_)^1.1^, according to the experimental observations reported for the case of the critical amplitude *A*_c_ [49].

When force curves were collected in contact mode, cantilevers with resonance frequencies *f*_0_ ≈ 15 kHz, spring constant *k* ≈ 0.2 N/m and *R* ≈ 10 nm were used (Nanosensors PPP-CONTR). The cantilever spring constant was calibrated in situ using the thermal noise method implemented in the MFP-3D microscope and the deflection in volts converted to deflection in nanometers by means of sensitivity calculations on dry CaF_2_ crystals.

Thin slices of CaF_2_ single crystals a few millimeters thick (Crystal GmbH, Berlin, Germany) were cleaved parallel to the (111) plane [40] in ambient conditions and kept under low humidity conditions before experiments. A low humidity level (RH < 10%) was achieved by mounting the samples on a cooler/heater holder for environmental control in the MFP-3D microscope and circulating dry nitrogen for 30 min after positioning the AFM head on top of the sample. Then the nitrogen flow was suspended and the cantilevers *k*, *f*_0_ and *Q* calibrated. AFM images of 2 × 2 µm^2^ were recorded before collecting APD curves in order to verify that no extended water patches were present on the surface.

When dry samples were needed, the crystals were kept at 120 °C for 30 min under nitrogen flow before calibrating the cantilevers.

In general, repeatedly scanning and/or collecting curves at ambient relative humidity RH ≈ 40% results in the formation of mesoscopic water patches on the surface of the crystals. Once the patches containing region was imaged (2 × 2 µm^2^) APD curves were collected on top of the patches. Images were collected at the lowest free amplitude (*A*_0_ = 9 nm) and with the highest amplitude set-point, in order to minimize sample deformation while scanning [41–42].

APD curves were converted into force curves by numerically integrating the Sader–Jarvis–Katan equation [38–39] in Matlab [53]. In this formalism, the conservative force *F*_ts_ vs *d*_min_ is recovered from variations in the oscillation amplitude (*A*) and in the frequency shift (Ω) that occur by decreasing the cantilever-surface separation (*z*). This is shown in Equation 1 where *d*_min_ is related to *z* and to *A* as: *d*_min_ ≈ *z* − *A*.

[1]
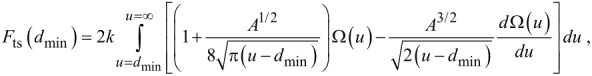


The normalized frequency shift Ω is derived from observables in AM-AFM. When *f* = *f*_0_, Equation 8 is obtained [38].

[8]
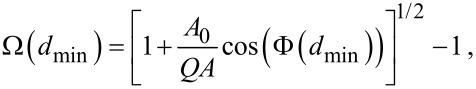


where Φ is the phase lag relative to the drive force, and *Q* is the quality factor due to dissipation with the medium.

The experimental APD curves cover the approach and the retract part during one cycle, with a drift smaller than 0.5 nm (see Supporting Information File 1, Figure S1). Only approach curves for which the cantilever–sample separation is decreased were employed to reconstruct the tip–sample force [27]. In this work raw (solid circles in grey) and smooth (continuous line in blue) data are reported for the reconstructed force curves [53].

The dissipated energy (*E*_diss_) and ΔΦ were also calculated for each APD curve vs *d*_min_. The energy dissipated per cycle was determined with the use of the expression derived by Cleveland et al. [31–32]:

[4]
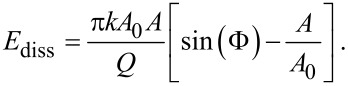


ΔΦ was calculated by subtracting the phase lag relative to the drive force, i.e., the experimental observable Φ, from the conservative angle Φ_cons_ defined as:

[5]
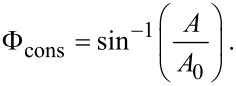


From Equation 5 it follows that if there is no energy dissipation, ΔΦ = 0.

### Numerical integration method

The standard (single mode) equation of motion [27,51] of the cantilever has been implemented in the programming language C and solved with the use of a standard Euler algorithm,

[9]
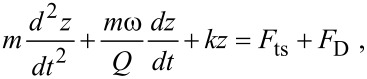


where ω is the angular drive frequency, the effective mass is *m* = *k*/ω^2^, *F*_D_ is the driving force and *F*_ts_ is the net tip–sample force. Typically, the drive frequency is set equal to the natural frequency ω_0_ since this leads to convenient simplifications. Furthermore, *z* is the position of the tip relative to its unperturbed equilibrium position.

The reconstruction of the conservative force and the determination of the energy dissipation have been carried out by implementing the expressions in Equation 1 and Equation 4 in Matlab [53]. The raw amplitude and phase data have been processed both when dealing with the experimental data and when dealing with the data from the numerical integration of the equation of motion with the same code implemented in Matlab. The expression in Equation 1 has been turned into a finite sum and numerically integrated in Matlab in both cases.

In the simulations three force profiles have been accounted for:

1) One where van der Waals (vdW) forces are present in the long range and repulsive forces are present in the short range. Long-range conservative vdW forces have been modeled, as it is customary in dynamic AFM theory [14,27,30,51], as:

[7]
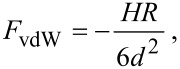


where H is the Hamaker constant.

Viscosity in the short range has been modeled with the Kelvin–Voigt model as [27,34–35]:

[10]
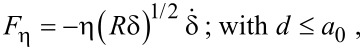


where η is the viscosity in Pascal·second, *a*_0_ is an intermolecular distance [14,27] and δ is the tip–sample deformation, i.e., δ = *a*_0_ − *d*. In this work η = 50 Pa·s throughout. In the short range the standard Derjaguin–Muller–Toporov (DMT) model of contact mechanics [54] has been employed to account for short range repulsion:

[11]
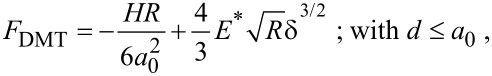


where E^*^ is the effective Young’s modulus that includes the elastic modulus of the tip and of the sample [14]. This profile is shown in Figure 4b.

2) The second profile corresponds to a linear decay in the long range force corresponding to the capillary interaction *F*_CAP_. *F*_CAP_ is written as:

[2]
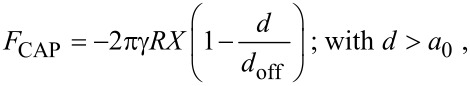


where γ is the surface energy and *X* is the average contact coefficient [3,14]. Equations 7, 8 and 9 have also been included. The resulting force profile is shown in Figure 3b.

For distances *d* < *d*_on_ hysteresis has been considered also and modeled simply as a difference in the Hamaker constant *H* during tip retraction relative to tip approach. In particular, *H*_retraction_ = 1.5·*H*_approach_. This term accounts for long-range dissipation in Figure 3b and could be identified with dissipation due to contact between the tip and the surface water layers. The relationship between γ and *H* is *H* = 24πγ(*a*_0_)^2^. For this profile *d*_on_ = 3 nm and *d*_off_ = 3.3 nm.

3) The third profile corresponds to a force curve displaying a plateau, such as the one shown in Figure 3c. In this case the van der Waals force in Equation 7 has been ignored, because it plays a minimal role as deduced by inspecting the experimental curves. Furthermore Equation 2 has been replaced by:

[3]
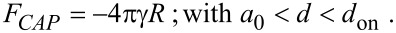


This profile is shown in Figure 3d. In the simulations *d*_on_ = 3 nm and *d*_off_ = 5 nm. The force has been assumed to remain constant and equal to that in Equation 3 in the *d*_on_/*d*_off_ region.

The common parameters in the simulations throughout this work are: *k* = 40 N/m, *f*_0_ = 300 kHz, *Q* = 450, *A*_0_ = 25 nm, *E*_tip_ = 120 GPa, *E*_sample_ = 1 GPa, *H* = 2.5 × 10^−20^ J, *R* = 8 nm.

## Supporting Information

File 1 APD curves, reconstructed force curves vs tip radius, effect of the choice of the “0” distance on the fitting parameters according to Equation 6.

## References

[1] García R, Magerle R, Perez R (2007). Nat Mater.

[2] Herruzo E T, Perrino A P, Garcia R (2014). Nat Commun.

[3] Sahagún E, García-Mochales P, Sacha G M, Sáenz J J (2007). Phys Rev Lett.

[4] Santos S, Barcons V, Font J, Thomson N H (2010). Nanotechnology.

[5] Santos S, Barcons V, Font J, Thomson N H (2010). J Phys D: Appl Phys.

[6] Gotsmann B, Seidel C, Anczykowski B, Fuchs H (1999). Phys Rev B.

[7] Sader J E, Jarvis S P (2004). Appl Phys Lett.

[8] Verdaguer A, Sacha G M, Bluhm H, Salmeron M (2006). Chem Rev.

[9] Amadei C A, Santos S, Pehkonen S O, Verdaguer A, Chiesa M (2013). J Phys Chem C.

[10] Amadei C A, Tang T C, Chiesa M, Santos S (2013). J Chem Phys.

[11] Katan A J, Oosterkamp T H (2008). J Phys Chem C.

[12] Wastl D S, Weymouth A J, Giessibl F J (2014). ACS Nano.

[13] Leroch S, Wendland M (2013). Langmuir.

[14] Barcons V, Verdaguer A, Font J, Chiesa M, Santos S (2012). J Phys Chem C.

[15] Heinz W F, Hoh J H (1999). Trends Biotechnol.

[16] Hernando-Pérez M, Miranda R, Aznar M, Carrascosa J L, Schaap I A T, Reguera D, de Pablo P J (2012). Small.

[17] Calò A, Reguera D, Oncins G, Persuy M-A, Sanz G, Lobasso S, Corcelli A, Payot-Augy E, Gomila G (2014). Nanoscale.

[18] Garcia-Manyes S, Redondo-Morata L, Oncins G, Sanz F (2010). J Am Chem Soc.

[19] Oncins G, Picas L, Hernández-Borrell J, Garcia-Manyes S, Sanz F (2007). Biophys J.

[20] Dols-Pérez A, Fumagalli L, Cohen-Simonsen A, Gomila G (2011). Langmuir.

[21] Dols-Pérez A, Fumagalli L, Gomila G (2014). Colloids Surf, B.

[22] Oncins G, Vericat C, Sanz F (2008). J Chem Phys.

[23] Stifter T, Marti O, Bhushan B (2000). Phys Rev B.

[24] Santos S, Gadelrab K, Font J, Chiesa M (2013). New J Phys.

[25] Rodrigues M S, Costa L, Chevrier J, Comin F (2014). J Appl Phys.

[26] Costa L, Rodrigues M S, Newman E, Zubieta C, Chevrier J, Comin F (2013). J Mol Recognit.

[27] Santos S, Amadei C A, Verdaguer A, Chiesa M (2013). J Phys Chem C.

[28] Giessibl F J (1997). Phys Rev B.

[29] Krüger D, Anczykowski B, Fuchs H (1997). Ann Phys.

[30] García R, Pérez R (2002). Surf Sci Rep.

[31] Cleveland J P, Anczykowsky B, Schmid A E, Elings V B (1998). Appl Phys Lett.

[32] Tamayo J, García R (1998). Appl Phys Lett.

[33] Santos S, Gadelrab K R, Barcons V, Stefancich M, Chiesa M (2012). New J Phys.

[34] Gadelrab K R, Santos S, Chiesa M (2013). Langmuir.

[35] Garcia R, Gómez C J, Martinez N F, Patil S, Dietz C, Magerle R (2006). Phys Rev Lett.

[36] Santos S, Gadelrab K R, Souier T, Stefancich M, Chiesa M (2012). Nanoscale.

[37] Gadelrab K R, Santos S, Souier T, Chiesa M (2012). J Phys D: Appl Phys.

[38] Katan A J, van Es M H, Oosterkamp T H (2009). Nanotechnology.

[39] Sader J E, Uchihashi T, Higgins M J, Farrel A, Nikayama Y, Jarvis S P (2005). Nanotechnology.

[40] Cardellach M, Verdaguer A, Fraxedas J (2011). Surf Sci.

[41] Santos S, Verdaguer A, Souier T, Thomson N H, Chiesa M (2011). Nanotechnology.

[42] Verdaguer A, Santos S, Sauthier G, Segura J J, Chiesa M, Fraxedas J (2012). Phys Chem Chem Phys.

[43] Butt H-J, Kappl M (2009). Adv Colloid Interface Sci.

[44] Yaminsky V V (1999). Colloids Surf, A.

[45] Zitzler L, Herminghaus S, Mugele F (2002). Phys Rev B.

[46] Verdaguer A, Weis C, Oncins G, Ketteler G, Bluhm H, Salmeron M (2007). Langmuir.

[47] Shinto H, Uranishi K, Miyahara M, Higashitani K (2002). J Chem Phys.

[48] Igor Pro 6.

[49] Santos S, Guang L, Souier T, Gadelrab K, Chiesa M, Thomson N H (2012). Rev Sci Instrum.

[50] Israelachvili J (1991). Intermolecular and Surface Forces.

[51] García R, San Paulo A (1999). Phys Rev B.

[52] García R, San Paulo A (2000). Ultramicroscopy.

[53] MATLAB R2010b.

[54] Derjaguin B V, Muller V M, Toporov Yu P (1975). J Colloid Interface Sci.

