# Supplementary material for: Capillary and van der Waals interactions on CaF2 crystals from amplitude modulation AFM force reconstruction profiles under ambient conditions
Source: Beilstein J Nanotechnol. 2015 Mar 25;6:809–19. doi: 10.3762/bjnano.6.84 (PMC4419597; doi:10.3762/bjnano.6.84)
Supplement: File 1 — APD curves, reconstructed force curves vs tip radius, effect of the choice of the “0” distance on the fitting parameters according to Equation 6. [file Beilstein_J_Nanotechnol-06-809-s001.pdf]

Supporting Information

for

Capillary and van der Waals interactions on CaF_2 crystals from amplitude modulation AFM force reconstruction profiles under ambient conditions

Annalisa Calò[1], Oriol Vidal Robles[1], Sergio Santos[2] and Albert Verdaguer*[1,3]

Address: [1]Institut Català de Nanociència i Nanotecnologia (ICN2), Campus UAB, Bellaterra, Barcelona, 08193, Spain; [2]Departament de Disseny i Programació de Sistemes Electrònics, Universitat Politècnica de Catalunya (UPC), Av. Bases 61, Manresa, Barcelona, 08242, Spain and [3]Consejo Superior de Investigaciones Científicas (CSIC), ICN2 Building, Bellaterra, Barcelona, 08193, Spain

Email: Albert Verdaguer* - averdaguer@cin2.es

* Corresponding author

APD curves, reconstructed force curves vs tip radius, effect of the choice of the "0" distance on the fitting parameters according to Equation 1.

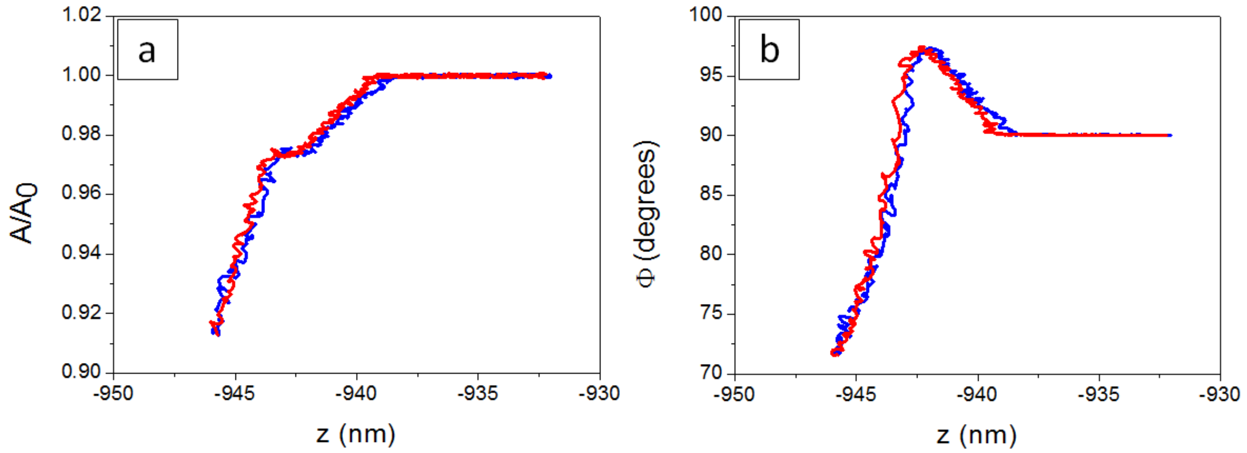

Figure S1: Normalized amplitude (a) and phase (b) curves vs tip–sample separation *z* exhibiting a smooth transition between the attractive and the repulsive regime. Free amplitude was 34 nm and the tip radius is estimated to be 10 nm within a 20% error.

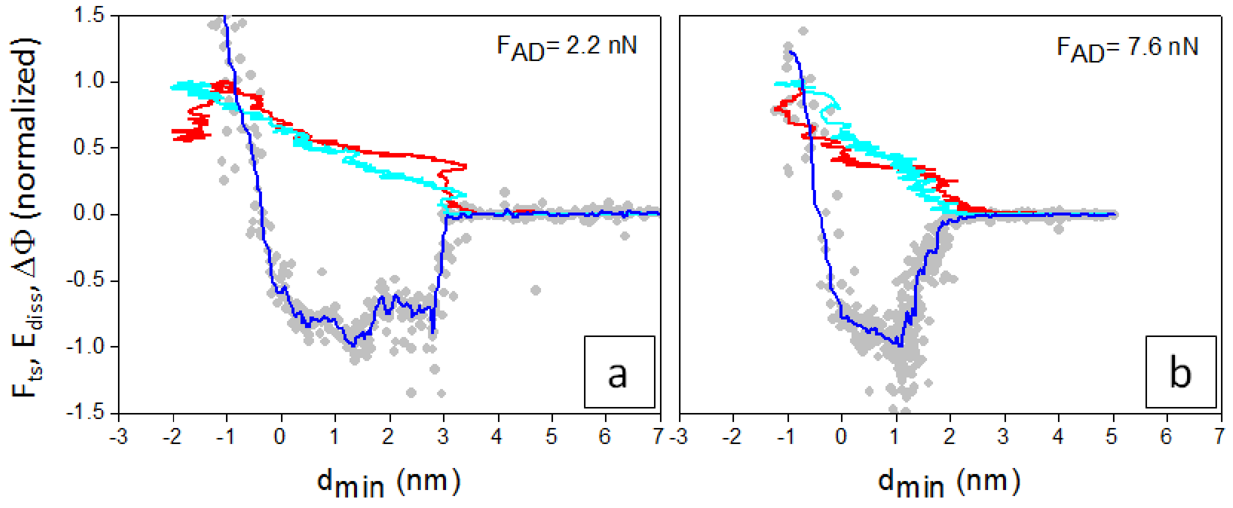

Figure S2: Reconstructed force curves for increasing tip radius, as it can be directly observed from the increase in the adhesion force F_{AD}. (a) Tip radius of about 10 nm. Force reconstruction over a water patch shows a well-defined plateau as in Figure 2b and Figure 3c. (b) Tip radius of about 40 nm. Force reconstruction over a water patch shows a linear decrease in the region of adhesion instead of a plateau. This behavior has been related to the presence of asperities in the tip [1] and in line with this assumption we only observed it for a relatively large tip radius.

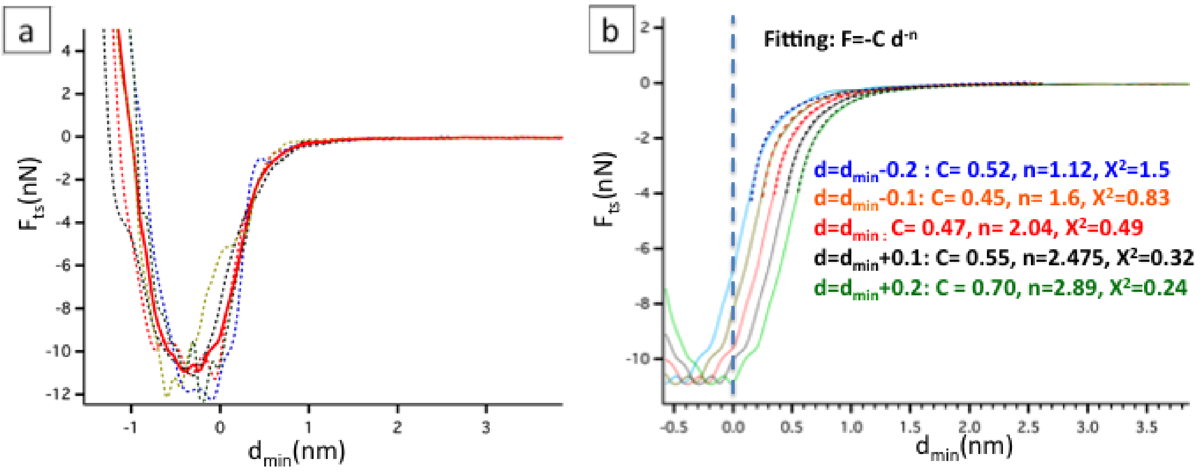

Figure S3: (a) Average curve (red line) from five reconstructed force curves taken at $A_0 = 80$ nm. In that case the position of the point at $d_{min} = 0$ in the x-axis for the fitting is more arbitrary. This point could indicate the distance at which the direct contact between adsorbed water layers still present on the tip and the sample begins. (b) Fitting of the initial curvature in the average curve for different positions of the "0". The fitting function was $F = -C \cdot d^{-n}$. The best fittings are for $n = 1.6$ and $n = 2.04$ according to χ^2 values. It is worthy to note that values of C remain stable around 0.5 for most of the fittings, indicating that even if the "0" is not properly chosen the effect on the C constant is minor.

References

1. Barcons, V.; Verdaguer, A.; Font, J.; Chiesa, M.; Santos, S. *J. Phys. Chem. C* **2012,** *116,* 7757–7766. doi:10.1021/jp2107395
